# Chasing the Dragon Syndrome: A Case of Toxic Leukoencephalopathy and Response to Intravenous Immunoglobulin

**DOI:** 10.7759/cureus.97318

**Published:** 2025-11-20

**Authors:** Piyush Puri, Jonathan Shadan, Michael Akhavan, Mehak Farrukh, Ramsha Durrani

**Affiliations:** 1 Internal Medicine, Icahn School of Medicine at Mount Sinai, Queens Hospital Center, New York, USA; 2 Medicine, New York Institute of Technology College of Osteopathic Medicine (NYITCOM), Old Westbury, USA

**Keywords:** coma, drugs, drug use, encephalopathy, heroin

## Abstract

Heroin inhalational leukoencephalopathy (HLE), also known as “chasing the dragon” syndrome, is a rare but severe neurological disorder associated with inhaling heroin vapors. It is characterized by progressive white matter damage and distinctive magnetic resonance imaging (MRI) findings, with no definitive treatment and a generally poor prognosis. We report the case of a 43-year-old man with a history of polysubstance abuse who presented with acute altered mental status. Initial investigations revealed aspiration pneumonia and a positive urine drug screen for cocaine and opioids. Magnetic resonance imaging (MRI) of the brain demonstrated diffuse, symmetrical T2-weighted fluid-attenuated inversion recovery (T2/FLAIR) hyperintensities in the white matter, sparing the deep gray nuclei, brainstem, and cerebellum, findings consistent with toxic leukoencephalopathy. Despite treatment with antibiotics, steroids, supportive care, and psychiatric management, his neurological condition deteriorated, requiring intensive care unit (ICU) admission and mechanical ventilation. Administration of intravenous immunoglobulin (IVIG) at a total dose of 2 g/kg over three days resulted in improvement in awareness and motor function. He was eventually discharged to a subacute rehabilitation facility. This case highlights the diagnostic complexity of HLE in the setting of multi-substance use, where imaging findings may overlap with other toxic encephalopathies. The observed clinical improvement following IVIG raises the possibility of a therapeutic role, warranting further investigation. Early recognition of (heroin inhalational leukoencephalopathy) HLE and its imaging findings is essential to initiate supportive interventions and explore emerging therapies.

## Introduction

“Chasing the dragon” is a term that appears to have first been seen in China in the 1920s. Because heroin was cheap but also impure, a mechanism to feel the effects of heroin without using an IV (intravenous) was created. A small quantity of powder is placed on aluminum foil on top of a heat source. When the powder starts to congeal and liquify, it emits a white vapor that is inhaled through a straw. The congealed powder or “dragon” is “chased” by the heat source underneath. This technique is widespread in Southeast Asia, Southwest Asia, and Western Europe [1].

Heroin inhalational leukoencephalopathy (HLE) is a rare toxic demyelinating disorder resulting from inhalation of heroin vapors, characterized by progressive white matter degeneration and distinctive MRI findings. Clinically, it presents with cerebellar dysfunction, motor impairment, and altered mental status, often leading to severe neurological disability or death.

Although the number of people who died from opioid overdose has decreased by 4% from 2022 to 2023, the number of deaths in 2023 was nearly 10 times higher than in 1999 [2]. Furthermore, the use of contaminated injection drug equipment is linked to increased transmission of infections like HIV and hepatitis C [3]. Therefore, we may see an increase in the prevalence of alternative methods of drug use, such as that seen with “chasing the dragon.”

## Case presentation

A 43-year-old male patient was admitted to the ED for an acute altered mental status. He has a history of cocaine use, two-pack-per-day smoking, type 2 diabetes, and hyperlipidemia. The patient was at baseline level the night before and was able to walk and talk normally. One day prior to his admission, he had come home from his job as a bus driver at 6 pm and went to his room at 8 pm with no changes in his disposition. In the morning, the patient’s mother went to see him in his room and found him unresponsive and drooling, but still moving all extremities. Narcan was administered prior to arriving at the ED without a change in mental status. He continued to be confused, unable to engage in conversation, and only opened his eyes to his name. After admission to the medicine unit, the patient was evaluated for potential causes of sepsis and encephalopathy.

During his initial admission, the patient’s vital signs were stable, with a temperature of 98.4 °F, heart rate of 86 bpm, blood pressure of 132/78 mmHg, respiratory rate of 18 breaths per minute, and oxygen saturation of 96% on room air. Neurological examination revealed confusion, limited verbal output, and delayed responses without focal motor deficits; pupils were equal and reactive, motor strength was 5/5 in all extremities, and deep tendon reflexes were symmetric.

At his second admission three weeks later, vital signs remained stable (temperature 99.1 °F, heart rate 92 bpm, blood pressure 138/80 mmHg, respiratory rate 20 breaths per minute, SpO_2_ 97% on room air). Neurologically, he exhibited slowed speech, impaired attention, and diminished spontaneous movement that progressed to mutism and decorticate posturing. Cranial nerve examination was grossly intact initially, but later became limited due to decreased responsiveness. These findings corresponded with radiologic evolution from an initially normal MRI to diffuse, symmetric T2-weighted fluid-attenuated inversion recovery (T2/FLAIR) white matter hyperintensities consistent with toxic leukoencephalopathy.

During evaluation, this patient tested positive for opiates and cocaine on his urine drug screen. He received antibiotics and fluids to treat aspiration pneumonia, the cause of his sepsis, and his mental status continued to improve. He was then discharged a few days later in stable condition, with instructions to follow up with the dependency clinic.

Three weeks later, the patient returned to the emergency department with weakness, decreased appetite, and insomnia that had persisted for a few days. His mother did not note any changes in his disposition after he was discharged from the hospital; however, he forgot his way home from work, which prompted her concern. The patient had slow speech on admission and was unable to answer questions properly. The next day, he was found to have a negative urinalysis, and there were no significant findings on his blood tests.

Electroencephalography (EEG) revealed cerebral dysfunction. His condition continued to decline as he progressed to an unresponsive and catatonic state, despite being able to walk and talk previously. A Glasgow Coma Scale (GCS) score <8 prompted a rapid response. Although the patient’s vital signs were stable, he was admitted to the ICU.

**Figure 1:**
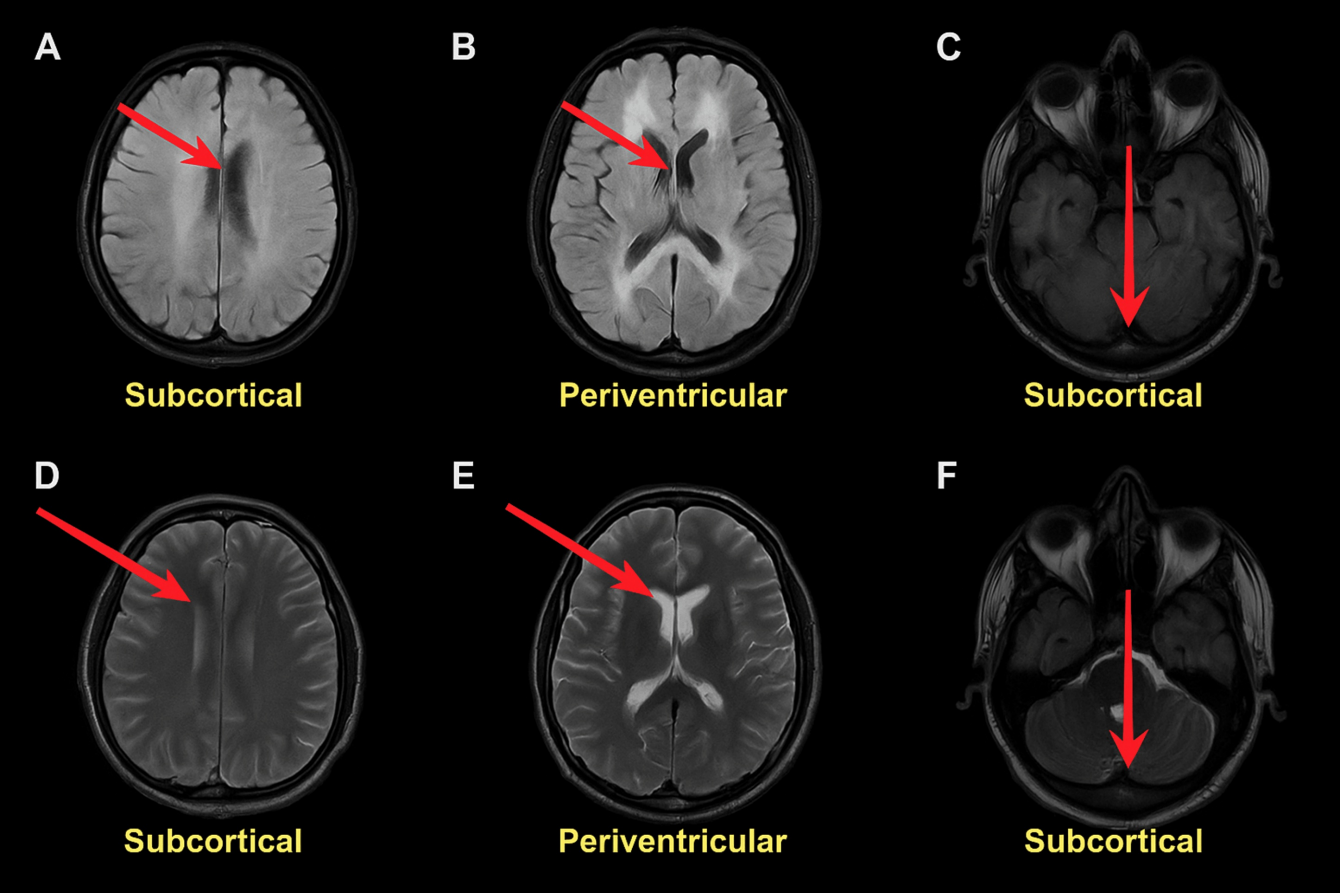
MRI brain Axial brain MRI demonstrating diffuse, symmetric white-matter hyperintensities (red arrows) involving the subcortical (A, C, D, F) and periventricular (B, E) regions on T2/FLAIR and T2-weighted sequences. The deep gray nuclei, brainstem, and cerebellar nuclei are spared. These imaging findings are typical of toxic leukoencephalopathy secondary to heroin vapor inhalation (“chasing the dragon”). T2/FLAIR: T2-weighted fluid-attenuated inversion recovery

Shortly after, the patient arrived at the medical intensive care unit (MICU) intubated and minimally sedated with propofol. Neurology believed that the patient may have been in status epilepticus, so they placed him on video electroencephalography (VEEG). VEEG showed no epileptiform discharges, seizures, or focal abnormalities; however, the background was consistent with moderate diffuse cerebral dysfunction. MRI of the brain with and without contrast performed on the same day demonstrated diffuse, bilateral, symmetric cerebral white matter T2/FLAIR hyperintensity without enhancement or diffusion restriction, sparing the deep gray nuclei, brainstem, and cerebellum, findings most consistent with toxic leukoencephalopathy (Figure 1).

The patient also experienced episodes of tachypnea, fever, hypertension, and diaphoresis with associated decorticate posturing. After consulting with neurology, the patient was started on vancomycin, ceftriaxone, ampicillin, and acyclovir for coverage of meningitis. A second lumbar puncture was performed one week after the first and was unremarkable. Seven days of pulse steroid therapy (Solu-Medrol) were administered, but no improvement was observed. Autonomic storming episodes continued, with a significant episode of storming with the heart rate in the 150s - 170s, respiratory rate (RR) in the 30s, febrile to 101.1 ºF with diaphoresis. The patient was consistently febrile with increased WBCs and was started on vancomycin and meropenem for a possible urinary tract infection (UTI) and was downtriaged to medicine.

The patient arrived at the medicine floor hemodynamically stable. He was treated with a trial of IVIG, which improved his attentiveness/alertness and movement of his extremities. He was seen by Psychiatry for a trial of Ativan, which did not improve his symptoms. A third lumbar puncture was performed about a month after the second or further evaluation of autoimmune etiologies. The CSF protein level was elevated, and the monocyte count was low. A percutaneous endoscopic gastrostomy (PEG) tube was placed, and a speech language pathologist (SLP) performed a fiberoptic evaluation of swallowing. The SLP recommended starting a thin/liquid diet with the plan to advance the diet as tolerated, with precautions for aspiration. A Passy-Muir valve was also provided to the patient for use under strict supervision. The patient was discharged approximately two months after admission to subacute rehabilitation and was referred to ENT (Ear, Nose & Throat). 

The patient’s neurological decline occurred approximately three weeks after his last known heroin use, coinciding with a period of abstinence confirmed by a negative urine drug screen. Initial imaging was unremarkable, but subsequent MRI demonstrated diffuse, symmetric white matter T2/FLAIR hyperintensities consistent with toxic leukoencephalopathy. Clinically, this evolution from mild confusion to akinetic mutism and posturing paralleled the radiologic progression, underscoring the delayed yet progressive nature of HLE despite cessation of exposure (Table 1).

**Table 1:** Clinical timeline of key events, interventions, and outcomes EEG: electroencephalography; MRI: magnetic resonance imaging; FLAIR: fluid-attenuated inversion recovery; IVIG: intravenous immunoglobulin; PEG: percutaneous endoscopic gastrostomy

Timeframe/hospital day	Major findings and interventions	MRI findings	Clinical response/outcome
Day 0 (initial admission)	Presented with altered mental status and aspiration pneumonia. Treated with antibiotics and supportive care.	MRI not performed.	Gradual improvement; discharged in stable condition.
~3 weeks post-discharge (readmission)	Returned with confusion, weakness, and insomnia; urine drug screen negative for opioids.	Diffuse, symmetric T2/FLAIR white matter hyperintensities sparing the deep gray nuclei, brainstem, and cerebellum—findings consistent with toxic leukoencephalopathy.	Progressive neurological decline with slowed speech and diminished motor response.
ICU course (week 1 of readmission)	Intubated for decreased responsiveness. Empiric therapy for meningitis/encephalitis (vancomycin, ceftriaxone, ampicillin, acyclovir). EEG showed diffuse cerebral dysfunction without seizures.	Repeat MRI unchanged.	No clinical improvement; developed autonomic storming and decorticate posturing.
Hospital day 14–21	Received pulse corticosteroid therapy (methylprednisolone × 7 days).	No radiologic improvement.	No significant neurological improvement.
Hospital day 25	IVIG therapy initiated (2 g/kg over 3 days).	No repeat MRI performed at that time.	Improved alertness and spontaneous extremity movement within days of therapy.
Following weeks (pre-discharge)	PEG tube placed; supportive rehabilitation and speech therapy initiated.	MRI not repeated prior to discharge.	Continued improvement in attentiveness and motor activity; discharged to subacute rehabilitation.

## Discussion

Heroin inhalational leukoencephalopathy (HLE), often referred to as "chasing the dragon" syndrome, is a neurological condition linked to heroin consumption. Specifically, it refers to inhaling the smoke that emits when heating heroin powder on aluminum foil. It is currently unknown why this method of heroin use causes HLE, but it is hypothesized that contamination in the heroin or a by-product of combustion could be involved [4].

Characteristic findings of HLE on brain biopsy include spongiform degeneration of the white matter and formation of vacuoles in the oligodendroglia and myelin sheaths [5]. Involvement of the cerebellum and posterior limb of the internal capsule with sparing of the anterior limb is frequently seen [1]. Widespread, symmetric T2/FLAIR hyperintensities are usually present within the white matter of the cerebellum and posterior cerebrum. The subcortical U-fibers, corticospinal tracts, and hippocampi are relatively spared. The brain MRI findings in our patient are consistent with these characteristic findings. Additionally, our patient has a history of drug abuse, and his urine drug screen was positive for opiates and cocaine.

The MRI findings discussed are considered pathognomonic for “chasing the dragon,” but other substances can mimic these findings. Cocaine toxicity can also present as diffuse symmetrical white matter changes on MRI [6]. Our patient also tested positive for cocaine on his first ED admission, so it remains unclear whether the observed changes were exclusively due to heroin inhalation or compounded by concomitant cocaine use. This overlap highlights the importance of a thorough toxicology screen and clinical correlation when interpreting such radiologic findings.

Although the positive cocaine screen presents a confounding factor, the clinical and MRI findings are more characteristic of heroin-induced leukoencephalopathy. Cocaine toxicity typically produces focal or patchy ischemic lesions involving the basal ganglia and subcortical white matter due to vasospasm or hypoxic-ischemic injury, whereas heroin vapor exposure causes diffuse, symmetric T2/FLAIR white matter hyperintensities with cerebellar and posterior cerebral predominance and sparing of the deep gray nuclei [1,6]. The delayed neurological decline despite abstinence is also a well-recognized feature of heroin-induced leukoencephalopathy [4]. These findings collectively support heroin vapor toxicity as the primary etiology, with concomitant cocaine use possibly exacerbating white-matter injury.

Additionally, MRI findings in chasing the dragon can progress despite being abstinent [6]. This was the case in our patient as well. The classic MRI findings were preceded by a few weeks of abstinence, as evident by his negative urine drug screen at his second visit to the ED.

There is currently no known treatment for HLE, but it is thought that antioxidants may provide some benefit in clinical and radiological findings. Vitamin A, C, E, zinc, coenzyme Q10, and selenium are usually recommended due to the neuroprotective effects of antioxidants in the brain [7,8]. The prognosis of HLE depends on the severity of symptoms. Patients with mild symptoms may have a mortality rate of 10%, while the mortality rate in patients with severe symptoms approaches 100% [9].

Clinically, the presentation of heroin-induced leukoencephalopathy varies among individuals, but the disease is often described as progressing through three distinct stages over the course of weeks to months. The initial stage is marked by cerebellar dysfunction, manifesting as dysarthria, ataxia, and motor restlessness. The onset of myoclonic jerks, choreoathetoid movements, and spastic paresis characterizes the intermediate stage. In the final stage, patients may develop akinetic mutism, extensor posturing, hyperthermia, and ultimately, death [10]. Previous studies have observed a disproportionately higher incidence of heroin-induced leukoencephalopathy among males and individuals of Asian descent, suggesting potential demographic susceptibility factors. Additionally, emerging evidence points to mitochondrial dysfunction in oligodendrocytes as a key contributor to white matter injury, linking impaired cellular energy metabolism directly to demyelination in affected patients [1,11]. Prior epidemiological studies have suggested a potential dose-response relationship in heroin-induced leukoencephalopathy, wherein individuals with a longer duration or higher frequency of heroin vapor inhalation were more likely to experience severe neurological outcomes. In the British Columbia case series by Buxton et al., all patients who died had a history of smoking heroin for at least three years, while one patient with a single exposure experienced only mild, non-hospitalized symptoms [4]. These findings support the hypothesis that cumulative exposure to either a combustion by-product or an intermittent contaminant may increase the risk and severity of white matter damage [4].

While pulse steroid, risperidone, and Ativan therapy did not improve our patient’s symptoms, a trial of IVIG therapy with 2 g/kg over three days (40 g q24 for three days) showed improvement in our patient's attentiveness/alertness as well as spontaneous extremity movement.

Although no established treatment exists for heroin-induced leukoencephalopathy, our patient demonstrated neurological improvement after IVIG (2 g/kg over three days) following steroid failure. Similar improvement has been reported in cases of progressive multifocal leukoencephalopathy (PML) and PML-IRIS (immune reconstitution inflammatory syndrome) treated with IVIG, suggesting a possible immunomodulatory benefit. These observations warrant further investigation to determine the therapeutic potential of IVIG in toxic or inflammatory leukoencephalopathies [12,13].

## Conclusions

Heroin inhalational leukoencephalopathy (HLE), or “chasing the dragon,” is a rare toxic leukoencephalopathy linked to heroin vapor inhalation. It presents with characteristic MRI findings, especially in the cerebellum and posterior cerebrum. While no definitive treatment exists, our patient showed clinical improvement with intravenous immunoglobulin (IVIG) after failing standard therapies, suggesting a possible role for immunomodulation in select cases.
